# Supplementary material for: Uniaxial-strain control of nematic superconductivity in SrxBi2Se3
Source: Nat Commun. 2020 Aug 24;11:4152. doi: 10.1038/s41467-020-17913-y (PMC7445267; doi:10.1038/s41467-020-17913-y)
Supplement: Supplementary file 1 — Supplementary Information [file 41467_2020_17913_MOESM1_ESM.pdf]

Uniaxial-strain control of nematic superconductivity in $Sr_xBi_2Se_3$

Kostylev *et al.*

Supplementary Note

1 Temperature dependence of resistivity at zero field

In Supplementary Figure 1, we show the temperature dependence of the zero-field resistance of $Sr_{0.06}Bi_2Se_3$ at three different strain values. For these measurements, we used the applied current of 250 μA. The superconducting critical temperature (T_c) defined as the mid-point of the transition is 2.83 K for zero relative strain $\Delta\varepsilon_{xx}$, i.e. zero applied voltage to the piezo stacks. With increasing $|\Delta\varepsilon_{xx}|$, T_c tends to decrease (See Supplementary Figure 7). With compressive strain of $\Delta\varepsilon_{xx}$= −1.19%, T_c decreases weakly by about 12 mK.

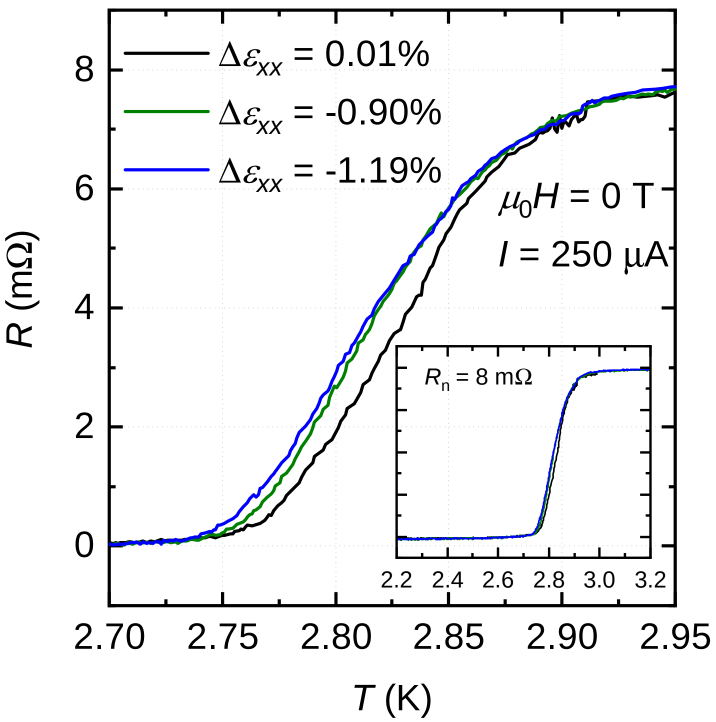

Supplementary Figure 1: Temperature dependence of resistance at zero field and at various applied strain for $Sr_{0.06}Bi_2Se_3$. These data were taken with the applied current I of 250 μA. In the inset, larger temperature range is shown to demonstrate the nearly T-independent resistance in the superconducting ($T <$ 2.7 K) and normal state ($T >$ 2.95 K) regions. R_n = 8 mΩ is the resistance in the normal-state. The main figure and its inset share the same vertical axis scale. Source data are provided as a Source Data file.

2 Raw *R* vs *B* data for all in-plane field angles

Here, we present a part of the raw magnetoresistance data, which are used to construct the color polar plot (Figs. 2a and b) and to evaluate H_{c2}. In Supplementary Figure 2, we show the in-plane magnetoresistance of $Sr_{0.06}Bi_2Se_3$ for field angles in the range of $-180°$ to $170°$ in steps of $10°$. It is clear that with compressive strain of $\Delta\varepsilon_{xx} = -1.19\%$, H_{c2} as well as the transition width decreases for $H \parallel x$ ($\phi_{ab} = 0°$ and $-180°$). Similar strain effect is also observed for the angles corresponding to the large H_{c2} direction of the minor domains (i.e. $\phi_{ab} = \pm30$ and $\pm150°$).

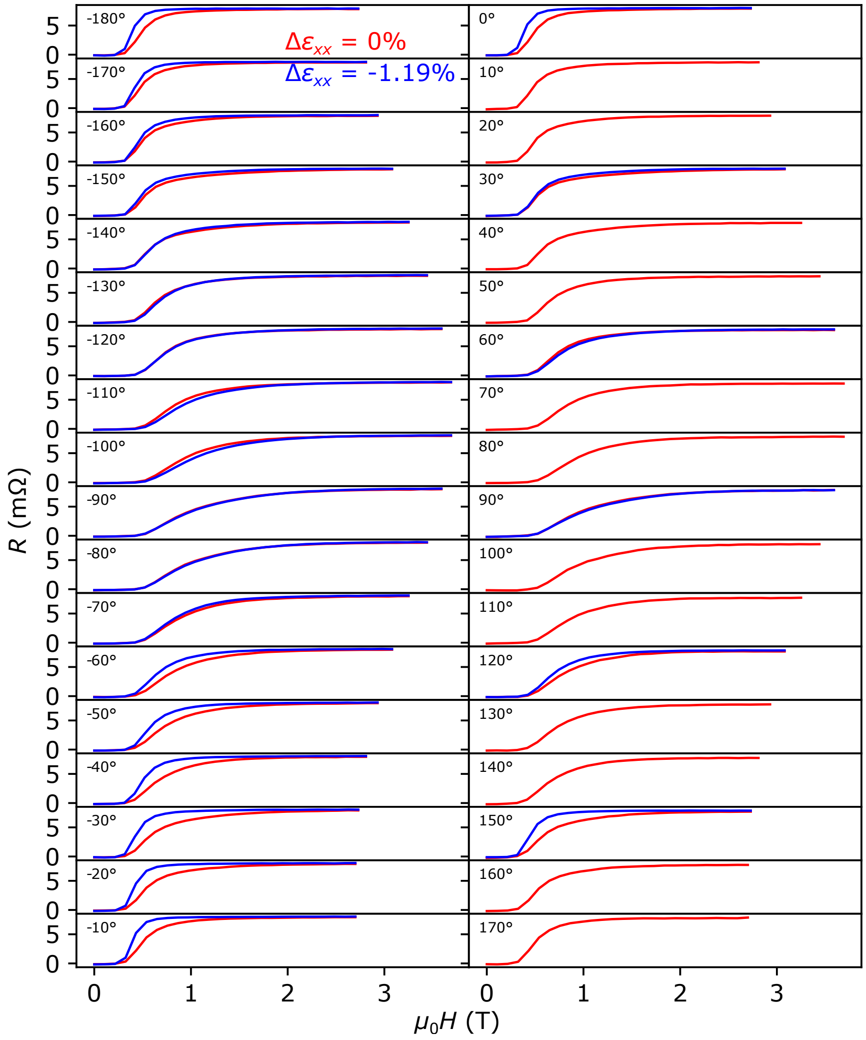

Supplementary Figure 2: Magnetoresistance for various in-plane field angles. The red and blue curves correspond to zero applied strain ($\Delta\varepsilon_{xx} = 0$) and compressive strain ($\Delta\varepsilon_{xx} = -1.19\%$), respectively. Source data are provided as a Source Data file.

3 Irreversible limit of the deformation

The strain range discussed in the Main Paper (i.e. $-1.5\% < \Delta\varepsilon_{xx} < +0.6\%$) is in the elastic deformation regime as evidenced by the reversible change of H_{c2} (Fig. 3). To support this claim, we sought for a border between the elastic and plastic regimes by applying stronger strains. Indeed, as shown in Supplementary Figure 3, we found that, after applying a sufficiently large compressive strain, the sample's electrical properties change irreversibly. After applying a large compressive strain of $\Delta\varepsilon_{xx} = -2.18\%$, the normal-state resistance increased by about 2 mΩ and T_c shifts down to about 2.4 K ($\Delta T_c \sim -0.4$ K), although T_c can be increased again by reducing the applied current from 250 μA down to 50 μA. The shift of the normal state resistance persisted after releasing the strain. The likely explanation is that formation of microcracks in the sample increases overall resistance and results in a Josephson-junction-like structure, which has much smaller critical current than bulk.

We also consider the Joule heating effect as the origin of the current-dependent T_c observed in this highly compressed sample. The Joule heating caused by the 2-mΩ increase of resistance is evaluated to be 0.005 nW for $I = 50$ μA and 0.125 nW for $I = 250$ μA. If the change in T_c is solely due to Joule heating, the sample temperature must be increased by 0.48 K with the 0.12-nW heating power. To achieve such a large temperature increase with a tiny heating power, the thermal conductance between the sample and the thermometer must be as small as 0.25 nW K^{-1}. Considering the situation that there are multiple and strong thermal connections between the sample and thermometer, through the four gold wires for resistivity measurements or through the uniaxial-strain device that are made of titanium, such a small thermal conductance is unrealistic.

Joule heating in the sample before reaching the deformation limit is discussed in Supplementary Note 12.

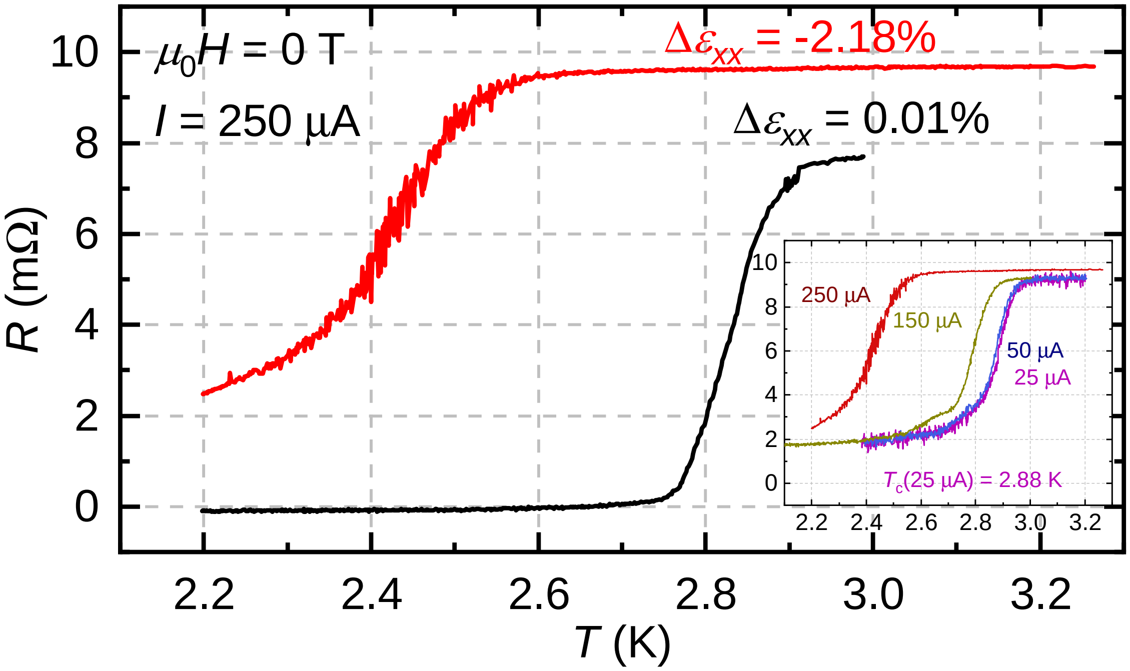

Supplementary Figure 3: Zero-field resistance versus temperature before and after irreversible change. The black and red curves correspond to zero applied strain and large compressive strain, respectively. (inset) Resistivity curves measured with various currents after the irreversible change. The critical temperature returns close to the original value (T_c = 2.88 K) by decreasing applied current down to 50 μA. Source data are provided as a Source Data file.

4 H_{c2} criteria

In order to explain H_{c2} evaluated in this work, we show in Supplementary Figure 4 an example how H_{c2} is determined from magnetoresistance curves using the criteria in the ratio between the resistance R and its normal-state value R_n. The process is to first decide on a criterion value of R/R_n, e.g. 50%. Then the $R(H)$ curve is linearly interpolated in-between points to determine the precise value of the magnetic field at which R/R_n reaches the criterion value: this value is taken to be H_{c2} of that criterion. In this work, we employ criteria R/R_n ranging from 5% to 95% to carefully examine the strain effect on nematic superconductivity.

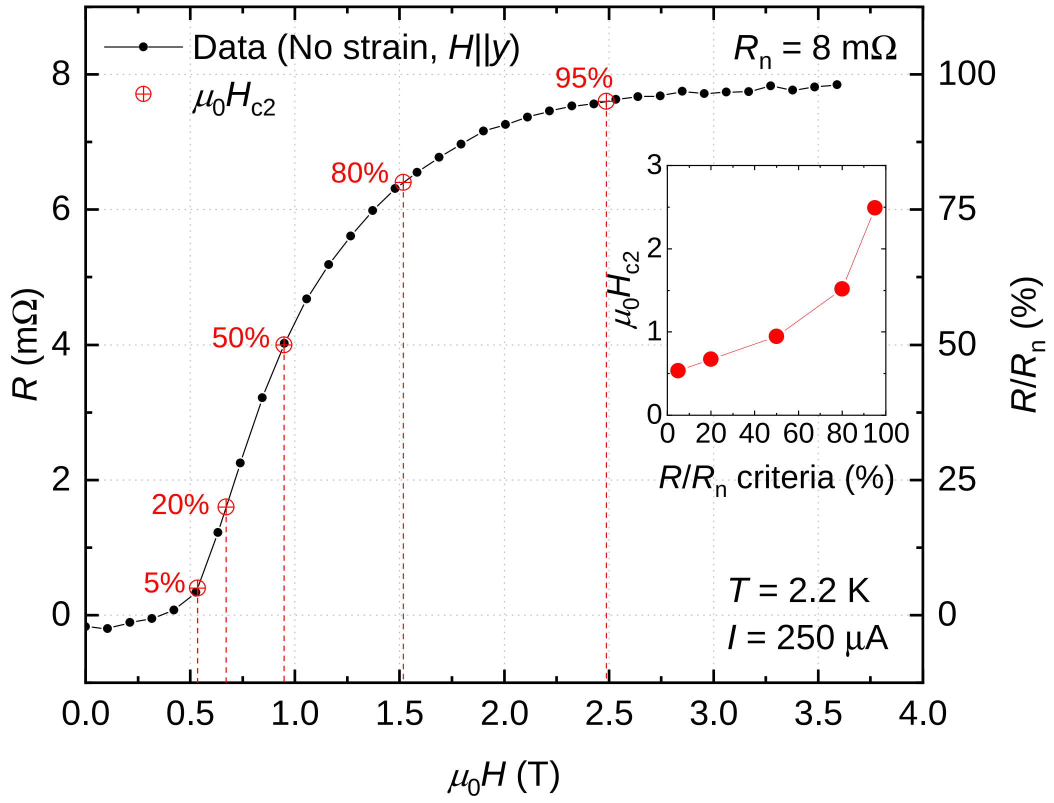

Supplementary Figure 4: Methodology for determining the upper critical field H_{c2} from magnetoresistance data. The data were taken under $\Delta\varepsilon_{xx} = 0\%$ and for $H \parallel y$ ($\phi_{ab} = -90°$). The red percentage labels indicate the R/R_n criteria used, where R_n is the normal-state resistance. The red circle and the corresponding vertical dotted line indicate the resistance value at the criteria and the corresponding field value (to be used as H_{c2}), respectively. (Inset) H_{c2} as a function of the R/R_n criteria. Source data are provided as a Source Data file.

5 Reproducibility of the strain control of nematic superconductivity

It is important to show that the strain control of the nematic superconductivity is reproducibly observed in other samples. In Supplementary Figure 5, we show the resistance and upper critical field (H_{c2}) of another sample (now referred to as Sample #2) of $Sr_{0.06}Bi_2Se_3$. The strain dependence of $H_{c2} \parallel x$ of Sample #2 is qualitatively similar to that of Sample #1, the sample that is mainly discussed in this Letter: a decreasing trend of $H_{c2} \parallel x$ with compressive strain. The strain effect is less significant in this sample, likely because the sample is already in a nearly single-domain state without the external strain. Indeed, in Supplementary Figure 6, the contour plot of magnetoresistance as functions of the polar and azimuthal field angles, we can see that the six-fold behavior due to minor domains is rather weak in Sample #2, compared with the similar plot of Sample #1 (Supplementary Figure 9b).

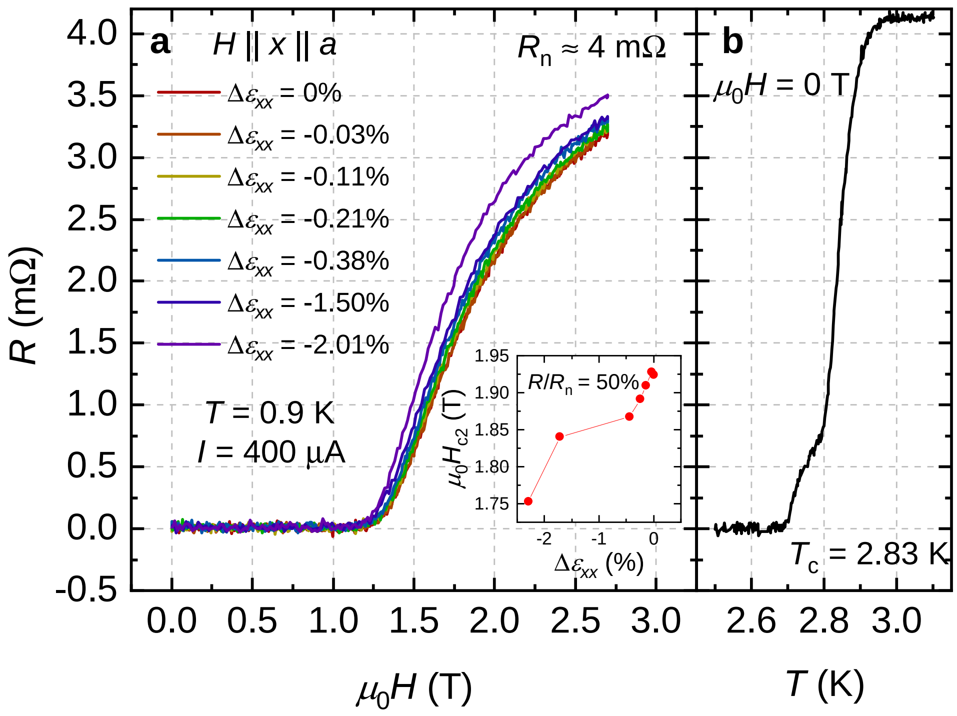

Supplementary Figure 5: Reproducibility of the strain control of nematic superconductivity of Sample #2. **a**, Magnetoresistance measured at 0.9 K and for $H \parallel x$ under various strains. The upper critical field evaluated from these curves (using the criteria $R/R_n = 50\%$) is shown in the inset as a function of $\Delta\varepsilon_{xx}$. Note that with increasing compressive strain the upper critical field tends to decrease. **b**, Zero-field temperature dependence of the resistance. For this sample, T_c evaluated at 50% of the transition is 2.83 K, which is very close to that of Sample #1. Source data are provided as a Source Data file.

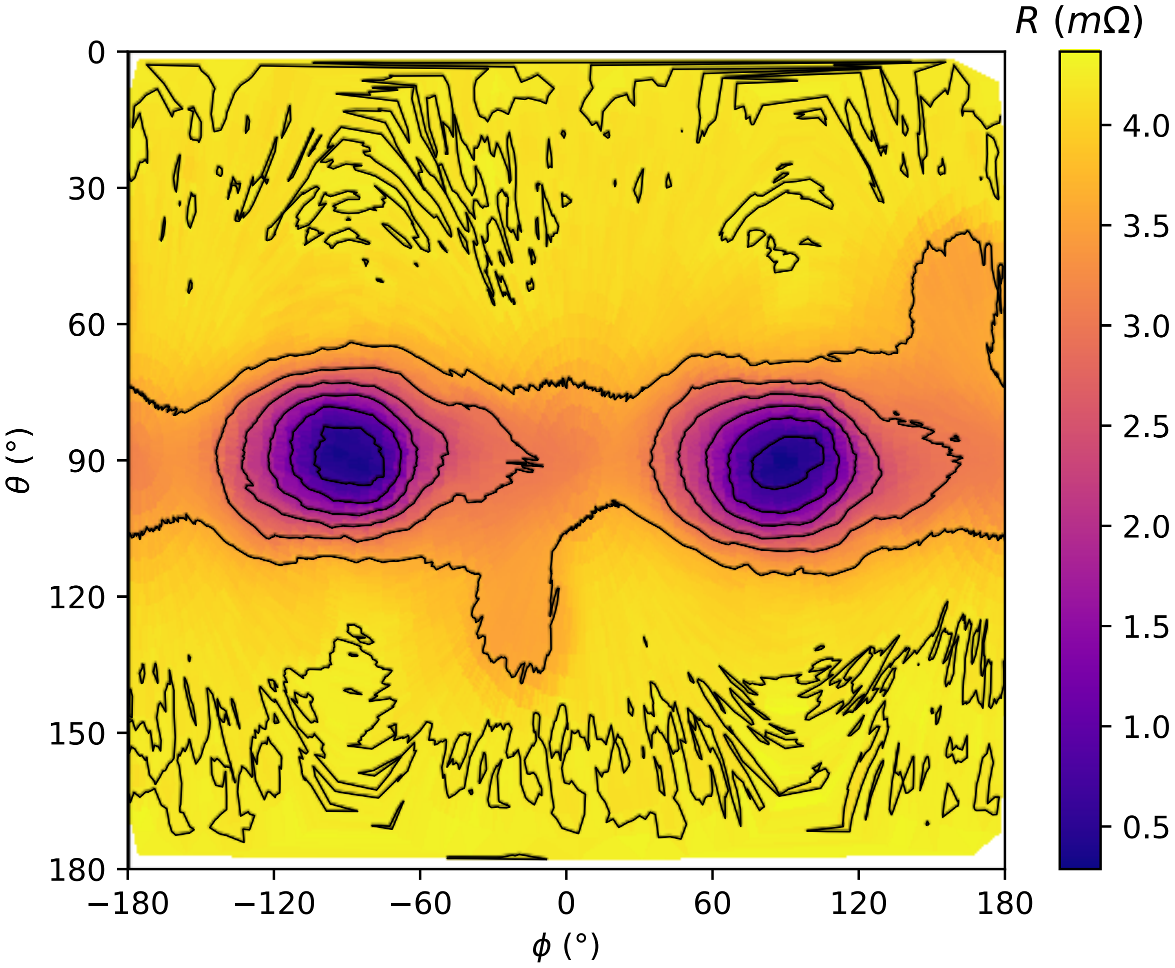

Supplementary Figure 6: Polar and azimuthal magnetic field dependence of resistance of Sample #2 at zero strain. The two-fold nematic SC component in the basal plane ($\theta = 90°$) is clearly seen. Notice that the contours around the purple region have oval shape, nearly free from dips at $\phi_{ab} = \pm30$ and $\pm150°$. This fact indicates that contributions from minor domains are much weaker in this sample than in Sample #1. The data here were taken at $\mu_0 H = 2.7$ T, $T = 0.9$ K, and $I = 400\,\mu$A. Source data are provided as a Source Data file.

6 Strain dependence of T_c

To see the strain dependence of superconducting properties other than the upper critical field, we show in Supplementary Figure 7 the dependence of the superconducting critical temperature T_c on the applied strain at zero field. Here, T_c is defined as the midpoint of the transition. We find a decreasing trend of T_c with compressive strain but the overall change is less than 1%. The change in T_c may be due to a change in the density of states, as reported in the hydrostatic-pressure study of $Sr_{0.06}Bi_2Se_3$[1].

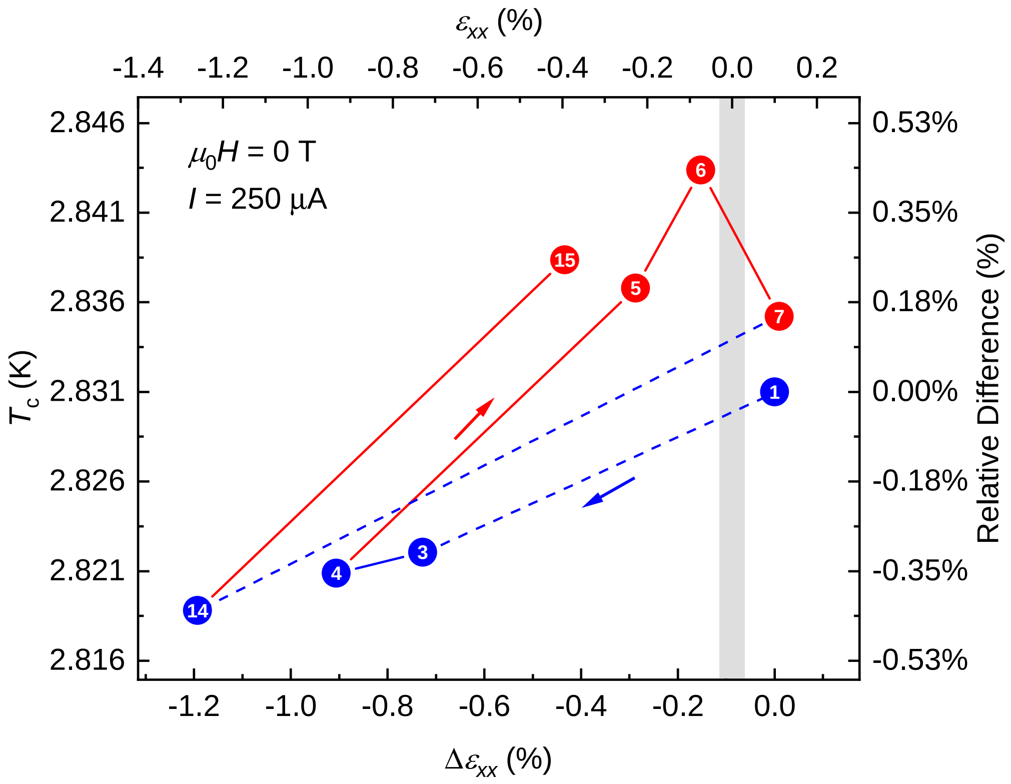

Supplementary Figure 7: Superconducting critical temperature versus applied strain at zero field. The numbers in the data points indicate the order of the measurements. The lines also indicate the measurement order. Among them, the dotted lines indicate that the measurement sequence number increases by more than 1, because zero-field resistance measurement was not performed between these measurement sequences. The blue and red data points indicate the cases that the measurement was performed after a decrease and increase in applied strain, respectively. The relative difference in the right vertical axis is defined as the change in T_c from the zero strain T_c (2.831 K). Source data are provided as a Source Data file.

7 Rotation of the nematic direction by applied uniaxial strain based on the Ginzburg-Landau theory

The Ginzburg-Landau (GL) free energy on the coupling between the nematic superconductivity and a uniaxial strain under existence of a pre-existing symmetry-breaking field (SBF) is given by[2]

$$f_{\mathrm{SB}} = (g\vec{\varepsilon} + g_0\vec{\varepsilon_0}) \cdot \vec{S}, \tag{1}$$

where $\vec{\varepsilon}$ is the applied strain vector, $\vec{\varepsilon_0}$ is the pre-existing SBF vector, $\vec{S}$ is the director of the nematic superconductivity, and g and g_0 are the coupling constants. Here we only consider the lowest-order coupling terms in order to keep simplicity. The strain and SBF vectors are expressed as

$$\vec{\varepsilon} = \begin{pmatrix} \varepsilon_{xx} - \varepsilon_{yy} \\ -2\varepsilon_{xy} \end{pmatrix} = U \begin{pmatrix} \cos(-2\phi) \\ \sin(-2\phi) \end{pmatrix}, \tag{2}$$

and

$$\vec{\varepsilon_0} = U_0 \begin{pmatrix} \cos(-2\phi_0) \\ \sin(-2\phi_0) \end{pmatrix}, \tag{3}$$

where U is the magnitude of applied anisotropic strain, U_0 is the magnitude of of the pre-existing SBF, ϕ is the angle of the strain within the basal plane of Bi_2Se_3, and ϕ_0 is the angle of the pre-existing SBF. The nematic SC order parameter is expressed as

$$\vec{\eta} = \begin{pmatrix} \eta_x \\ \eta_y \end{pmatrix} = \eta \begin{pmatrix} \cos\phi_\eta \\ \sin\phi_\eta \end{pmatrix}. \tag{4}$$

With this notation, the $\vec{S}$ vector has the form

$$\vec{S} = \begin{pmatrix} |\eta_x|^2 - |\eta_y|^2 \\ -2\eta_x\eta_y \end{pmatrix} = \eta^2 \begin{pmatrix} \cos(-2\phi_\eta) \\ \sin(-2\phi_\eta) \end{pmatrix}. \tag{5}$$

Substituting (5), (2), and (3) into (1), then (1) simplifies to

$$f_{\mathrm{SB}} = \eta^2[gU\cos(2(\phi_\eta - \phi)) + g_0U_0\cos(2(\phi_\eta - \phi_0))]. \tag{6}$$

The nematicity direction ϕ_η is chosen such that the free energy is minimized (i.e. $df_{\mathrm{SB}}/d\phi_\eta = 0$ and $d^2f_{\mathrm{SB}}/d\phi_\eta^2 > 0$):

$$\Delta\phi_\eta = -\frac{1}{2}\arctan\left(\frac{\sin(2(\Delta\phi_0))}{gU/g_0U_0 + \cos(2(\Delta\phi_0))}\right) + \frac{\pi}{2}k, \tag{7}$$

where $\Delta\phi_\eta \equiv \phi_\eta - \phi$ is the nematicity direction with respect to the applied strain direction, $\Delta\phi_0 \equiv \phi - \phi_0$ is the angle between the strain and the pre-existing SBF, and k is an integer chosen such that $d^2 f_{\text{SB}}/d\phi_\eta^2 > 0$ is satisfied.

The result of the above Supplementary Equation (7) is shown in Supplementary Figure 8. It is evident that, when the angle between the applied strain and the pre-existing SBF is orthogonal ($\Delta\phi_0 = 90°$), the nematic direction changes discontinuously when the applied strain term is equal to the pre-existing SBF term (i.e. $gU = g_0U_0$). When the pre-existing SBF is parallel ($\Delta\phi_0 = 0°$), the nematic direction changes suddenly when the applied strain term is equal and opposite to the pre-existing SBF term (i.e. $gU = -g_0U_0$). At intermediate angles, the direction of the nematicity changes continuously but rapidly until the ratio of the applied and pre-existing SBF, gU/g_0U_0, reaches about 1, after which it changes more gradually. This rotation is very likely the driving force of the nematic domain change under uniaxial compression observed in this work.

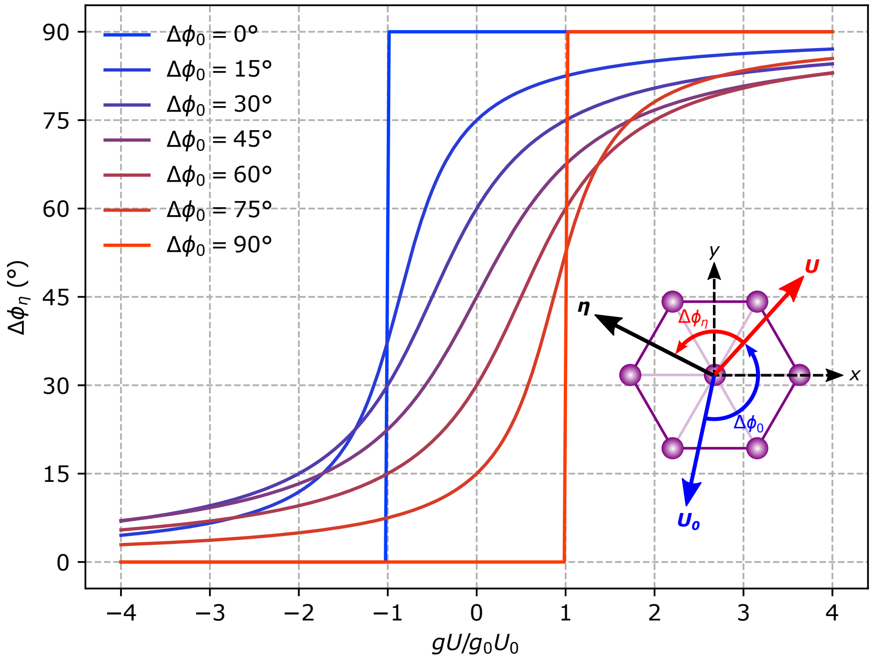

Supplementary Figure 8: Rotation of the nematic direction by applied uniaxial strain. The uniaxial strain with the magnitude U is applied at an angle $\Delta\phi_0$ from the pre-existing symmetry breaking field (strength U_0). $\Delta\phi_\eta$ is the angle relative to the axis of the applied strain. The inset shows the definitions of the axes and the symmetry breaking fields (U, U_0) and nematic director (η) with respect to the crystal structure in the *ab* plane.

8 Transformation between the laboratory and sample frames

In this section, we describe the procedure to determine the transformation relation between the sample and laboratory frames, to align magnetic fields accurately with respect to the crystalline axes.

In this work, the magnetic field was applied using a vector-magnet system, which consists of two orthogonal superconducting magnets: one pointing in the vertical direction and the other in the horizontal direction in the laboratory frame[3]. The polar and azimuthal angles of the magnetic field are indicated by $\theta_{\rm Lab}$ and $\phi_{\rm Lab}$, respectively. To know the transformation between the laboratory frame angles ($\theta_{\rm Lab}$ and $\phi_{\rm Lab}$) and the sample frame angles (θ and ϕ), we made use of the anisotropy in $H_{\rm c2}$. We first measured the angular magnetoresistance in the superconducting transition region, covering the full 4π solid angle of the magnetic field, as shown in Supplementary Figure 9a in the laboratory frame. Because $H_{\rm c2}$ of $\mathrm{Sr}_x\mathrm{Bi}_2\mathrm{Se}_3$ is smallest along the c axis[4], the field direction with the largest resistance is the c direction and the plane with relatively small resistance should be the ab plane. If this data is correctly transformed into the sample frame by using a 3×3 rotation matrix R, the former should be located at $\theta = 0$ or $180°$, and the latter should lie at $\theta = 90°$. Thus, our goal is to find such a matrix R.

In general, a vector in the laboratory frame $v_{\rm Lab}$ transforms to a vector in the sample frame v via:

$$v = R \cdot v_{\rm Lab}. \tag{8}$$

The vectors v and v_{Lab} are in Cartesian coordinates. The rotation matrix R can be decomposed into three elemental rotation matrices with the Euler angles α, β, and γ :

$$R = Z(\gamma)X(\beta)Z(\alpha), \tag{9}$$

which corresponds to the combination of a rotation by α about z axis, then a rotation by β about the rotated x axis, and then a rotation by γ about the rotated z axis. Note that the elemental rotation matrices are given as follows:

$$X(\omega) = \begin{pmatrix} 1 & 0 & 0 \\ 0 & \cos\omega & -\sin\omega \\ 0 & \sin\omega & \cos\omega \end{pmatrix} \tag{10}$$

$$Z(\omega) = \begin{pmatrix} \cos\omega & -\sin\omega & 0 \\ \sin\omega & \cos\omega & 0 \\ 0 & 0 & 1 \end{pmatrix}, \tag{11}$$

where ω is one of the Euler angles. Lastly, the sample frame vector v is converted from Cartesian to spherical coordinates defined by the two variables θ and ϕ. The basal plane ϕ_{ab} is given as ϕ on the plane of $\theta = 90°$.

When determining the Euler angles from the experiment, we first find the plane of low resistance (i.e. the ab plane) comes on the plane $\theta = 90°$ when we used the Euler angles $\alpha = -105°$ and $\beta = 88°$. To determine γ, we need to use the fact that the sample's x axis (one of the a axes) is roughly oriented along $\theta_{Lab} = 0°$, as described in Methods. This x axis should be transformed to $\phi = 0°$ in the $\theta = 90°$ plane after the γ rotation. This determines the last Euler angle γ to be 31°. With this combination of the Euler angles, the angular magnetoresistance is now transformed as shown in Supplementary Figure 9b, matching with the expectation explained above.

Finally, the magnetic field coordinate matrices expressed in the sample frame H_{Sample} and that in the laboratory frame H_{Lab} can be converted back and forth via the relation

$$H_{\mathrm{Sample}} = R \cdot H_{\mathrm{Lab}}. \tag{12}$$

As mentioned in Methods, the magnetic field presented in the Main Text are all expressed in the sample frame determined in this way.

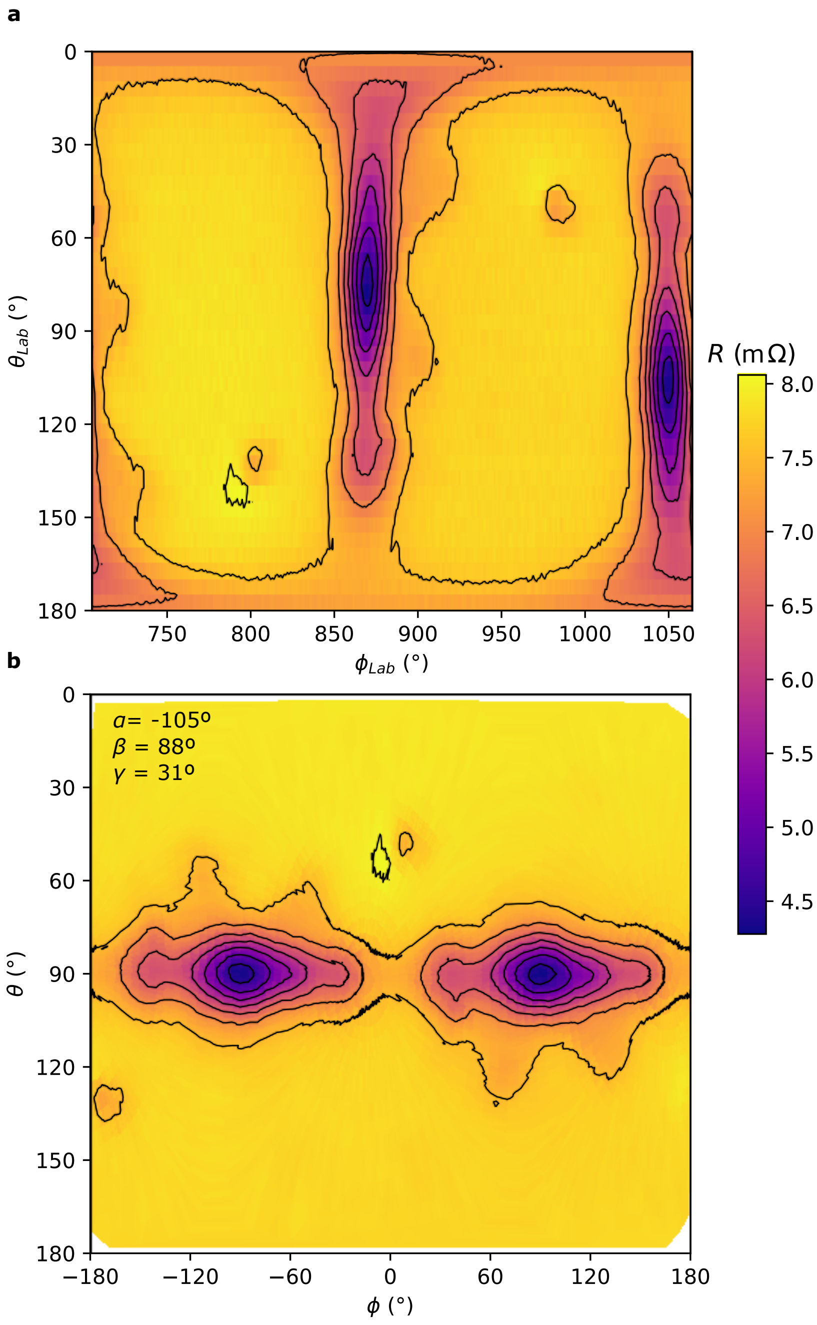

Supplementary Figure 9: Euler transform from laboratory to sample frame. **a**, Contour plot of the angular magnetoresistance plotted as functions of the azimuthal and polar field angles in the laboratory frame. The data were obtained at 2.2 K and 1 T. **b**, Same data plotted as functions of field angles in the sample frame, after rotating the data by using the Euler angles ($\alpha = -105°, \beta = 88°, \gamma = 31°$). Source data are provided as a Source Data file.

9 Angular magnetoresistance at zero applied strain

In this section, we show angular magnetoresistance covering the whole 4π solid angles of the field directions at zero applied strain, in order to demonstrate that the observed behavior is not due to the field misalignment.

In Supplementary Figure 10, we show the colour plots of the magnetoresistance as functions of the polar and azimuthal field angles, measured at different magnetic field strength and temperature at zero applied strain (i.e. $\Delta\varepsilon_{xx} = 0\%$). Evidently, for all cases the strong two-fold behavior along the ϕ direction due to the nematic superconductivity is seen. For low temperature and/or low field, most of the angles are largely in the superconducting state (corresponding to the dark-blue region), whereas for high temperature or high field only the regions with largest upper critical field remain in the superconducting state. From these data, we confirm that our alignment of the magnetic field to the crystal axis is quite accurate and field-misalignment effect is negligible.

In addition to the strong two-fold behavior, the data near the onset (the two bottom panels of Supplementary Figure 10) exhibit small anomalies at around $\phi = \pm 30^\circ$ and $\pm 150^\circ$. See that some contours have dips at these angles. These anomalies are due to the existence of nematic subdomains as discussed in the Main Text.

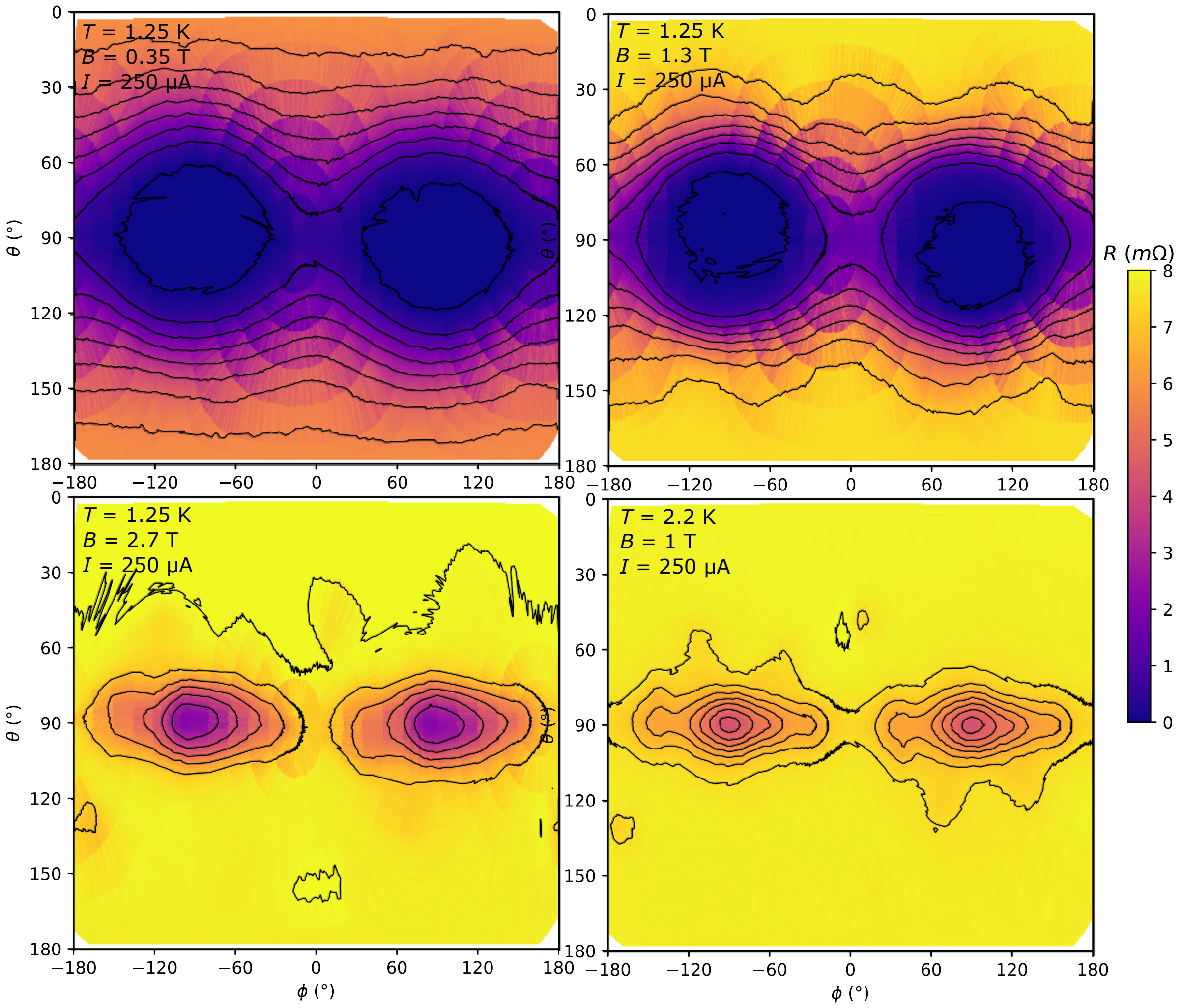

Supplementary Figure 10: Polar and azimuthal angle dependences of the magnetoresistance at zero strain for various magnetic-field and temperature conditions. The light yellow and dark blue regions correspond to normal state and superconducting state, respectively. Measurement conditions are indicated in the top-left corner of each panel. Source data are provided as a Source Data file.

10 Model simulation

In order to simulate the magnetoresistance and the upper-critical-field behavior under single and multiple nematic SC domains, we performed a model simulation. In this section, details of the simulation will be discussed. We should comment here that our model is independent of the choice of mechanism of finite resistivity appearing under finite magnetic field, since we made use of experimental data to model the resistivity behavior of one domain as described below. In addition, we used equations for classical circuit analysis, which is again independent of the choice of detailed mechanism of resistivity.

Magnetoresistance of each domain

Firstly, we have to define the magnetoresistance behavior of each domain. We assumed that the magnetoresistance of a single nematic SC domain obeys the following empirical equation:

$$\frac{R(H, \phi_{ab})}{R_{\mathrm{n0}}} = \left[1 + \left(\frac{H}{H_{\mathrm{c2}}(\phi_{ab})}\right)^{-h(H_{\mathrm{c2}}(\phi_{ab}))} (2^{1/s} - 1)\right]^{-s}, \tag{13}$$

where R_{n0} is the normal state resistance of the domain, ϕ_{ab} is the in-plane field angle, H_{c2} is the upper critical field (midpoint), s is an exponent determining the shape of the $R(H)$ curve around H_{c2}, and $h(H_{\mathrm{c2}})$ is another exponent introduced to depict the H_{c2}-dependent transition width. Notice that the coefficient $2^{1/s} - 1$ is a correction factor to make the right-hand side of Supplementary Equation (13) to 1/2 at $H = H_{\mathrm{c2}}(\phi_{ab})$. The functional form of the right-hand side of Supplementary Equation (13) is shown in Supplementary Figure 11.

For the actual simulation, we used the exponent $s = 2.03535$ and we employed an empirical relation $h(H_{\mathrm{c2}}) = -2.696 \times H_{\mathrm{c2}}(\phi_{ab})/H_{\mathrm{c2,max}} + 5.74248$ to reproduce the observed resistance behavior of the actual sample, in particular the H_{c2}-dependent broadening of the transition. Here, $H_{\mathrm{c2,max}}$ is the maximum H_{c2} within the ab plane. The angular dependence of H_{c2} is approximated by the anisotropic mass model:

$$H_{\mathrm{c2}}(\phi_{ab}) = \frac{H_{\mathrm{c2,max}}}{\sqrt{\cos^2(\phi_{ab} - \phi_0) + \Gamma^2 \sin^2(\phi_{ab} - \phi_0)}}, \tag{14}$$

where $\Gamma \equiv H_{\mathrm{c2,max}}/H_{\mathrm{c2,min}}$ is the anisotropy, and $H_{\mathrm{c2,min}}$ is the minimum H_{c2} given by $\phi_{ab} = \phi_0 \pm \pi/2$. The value of ϕ_0 defines the nematic superconducting domains: For the Y_n domain ($n = 0, 1, 2$), ϕ_0 is given by $\pi/2 + n\pi/3$. We found that $\Gamma = 3$ best reproduces the experimental data. Thus, this Γ value is used hereafter. Another important parameter, $H_{\mathrm{c2,max}}$ is set to 1 T unless explicitly mentioned, to reproduce the $R(H)$ curve at 2.2 K.

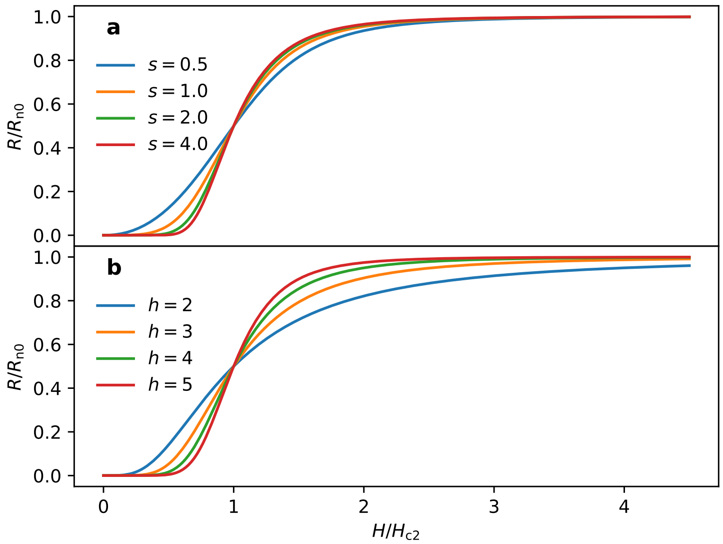

Supplementary Figure 11: Plot of Supplementary Equation (13), representing resistance versus applied magnetic field for a single domain, for varying parameters. **a**, For varying s where $h = 4.39$. **b**, For varying h where $s = 2.04$.

With these formulations and parameters, Supplementary Equation (13) exhibits functional forms shown in Supplementary Figure 12 in the case of $\phi_0 = \pi/2$ (Y_0 domain). Comparing these curves with the raw data shown in Fig. 1c, we can see that Supplementary Equation (13) well reproduces the observed magnetoresistance of the strained sample (corresponding to a single Y_0 domain state). Thus, the formulation described above should be valid for the simulation.

Circuit model of multiple domains

Next, we have to assume a certain circuit to model the distribution of domains. In Supplementary Figure 13, we present the electrical circuit model used to produce data in Fig. 4, consisting of a 3D network of twelve resistive elements R_{1a}, R_{1b}, ..., R_{2B}. The end-to-end total resistance of this circuit R_{total} is given by a certain function f:

$$R_{total}(H, \phi_{ab}) = f\left(R_{1a}(H, \phi_{ab}), R_{1b}(H, \phi_{ab}), ..., R_{2B}(H, \phi_{ab})\right), \tag{15}$$

which is determined by standard techniques of circuit analysis. The normal-state resistance $R_{\text{total,n}}$ of the net circuit is given by

$$R_{\text{total,n}} = R_{\text{total}}(H \to \infty) \tag{16}$$

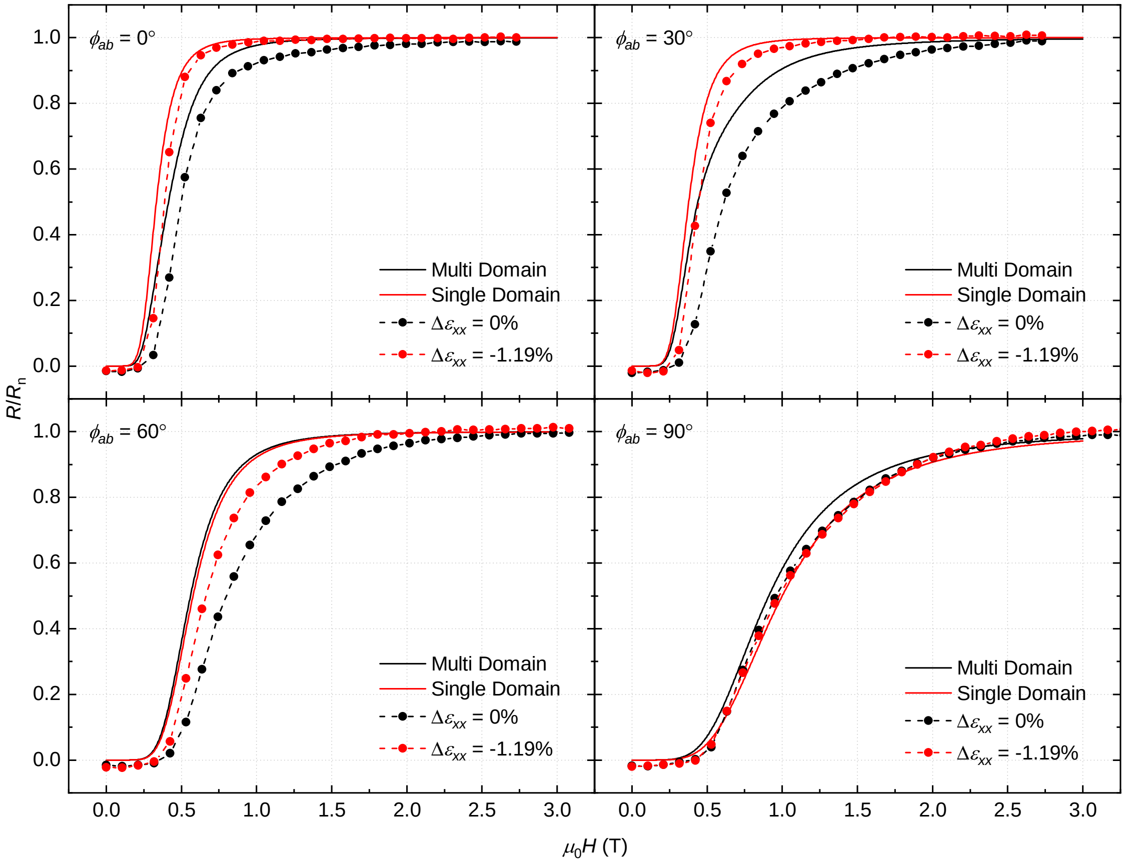

Supplementary Figure 12: Comparison between data and model for R/R_n vs $\mu_0 H$ at different in-plane angles. The angle of the magnetic field is shown by ϕ_{ab} in the top-left corner in each panel. The multi domain model is given by Supplementary Equation (15). The single domain model is given by Supplementary Equation (13). The nematic anisotropy used is $\Gamma = 3$. The maximum in-plane upper critical field is $\mu_0 H_{\text{c2,max}} = 1$ T. Source data are provided as a Source Data file.

For the multi-domain simulation presented in Fig. 4 of the Main Text, we assumed that $R_{1\text{a}}$ and $R_{2\text{B}}$ are Y_1 and Y_2 domains and the rest are Y_0 domains. For each domain, the normal-state resistance value R_{n0} is assumed to be the same. Magnetoresistance curves obtained for this multi-domain case are shown in Supplementary Figure 12, which captures features of the magnetoresistance of the unstrained sample (i.e. $\Delta\varepsilon_{xx} = 0$). For the single-domain simulation, we set

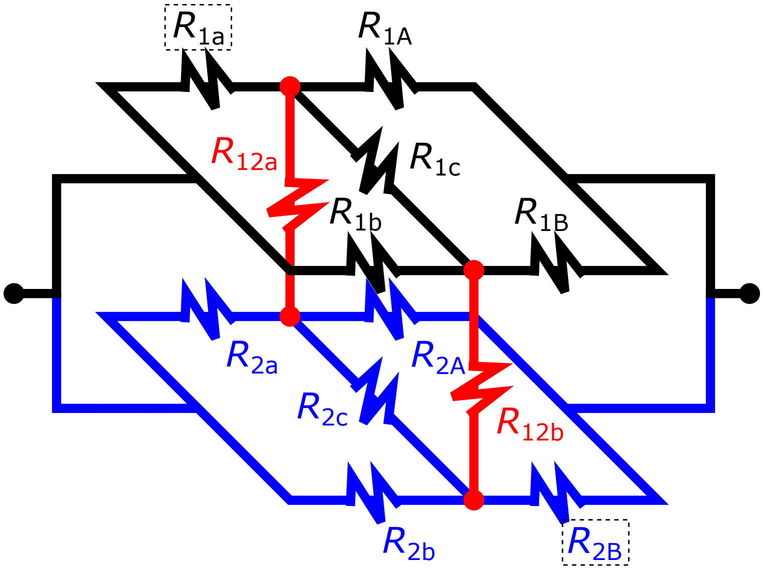

Supplementary Figure 13: Electrical circuit of a 3D network of resistive elements used for the simulation. The black and blue colored lines represent the top and bottom parts of the circuit, respectively. The red lines are the interconnecting paths between the top and bottom parts of the circuit. The elements marked by the dotted box are set to the minor domains in case of the multi-domain simulation.

all components to the Y_0 domain. We comment that, for the single domain case, $R_{\mathrm{total}}/R_{\mathrm{total,n}}$ is identical to R/R_{n0} of Supplementary Equation (13).

Illustrative explanation using a simpler model

In order to illustrate how the path of the current changes depending on the direction of the applied magnetic field, we show a simplified version of the above circuit in Supplementary Figure 14. When the direction of the applied magnetic field is parallel to the axis that has the largest H_{c2} for the dominant domain, Y_0 (Supplementary Figure 14a), then the Y_0 domain has lower resistance than the minor domains Y_1 and Y_2 and hence the current passes mostly through the Y_0 domains. If the field angle is aligned with the H_{c2} maximum of either the Y_1 or Y_2 domains (Supplementary Figure 14b or c), then the current will certainly pass through those domains. However, due to the configuration of the domains in the network, the current must pass through a Y_0 domain as well to reach the opposite end. This effect is what ensures that the minor domains have a relatively smaller influence on H_{c2} than the dominant domain except for the very vicinity of the onset of superconductivity. Hence we get the characteristic 6-fold in-plane H_{c2} with one of the 2-fold H_{c2} being relatively larger than the other near the onset (95 or 80% criteria H_{c2}) but purely two-fold

behavior close to zero resistance state (20 or 5% criteria H_{c2}).

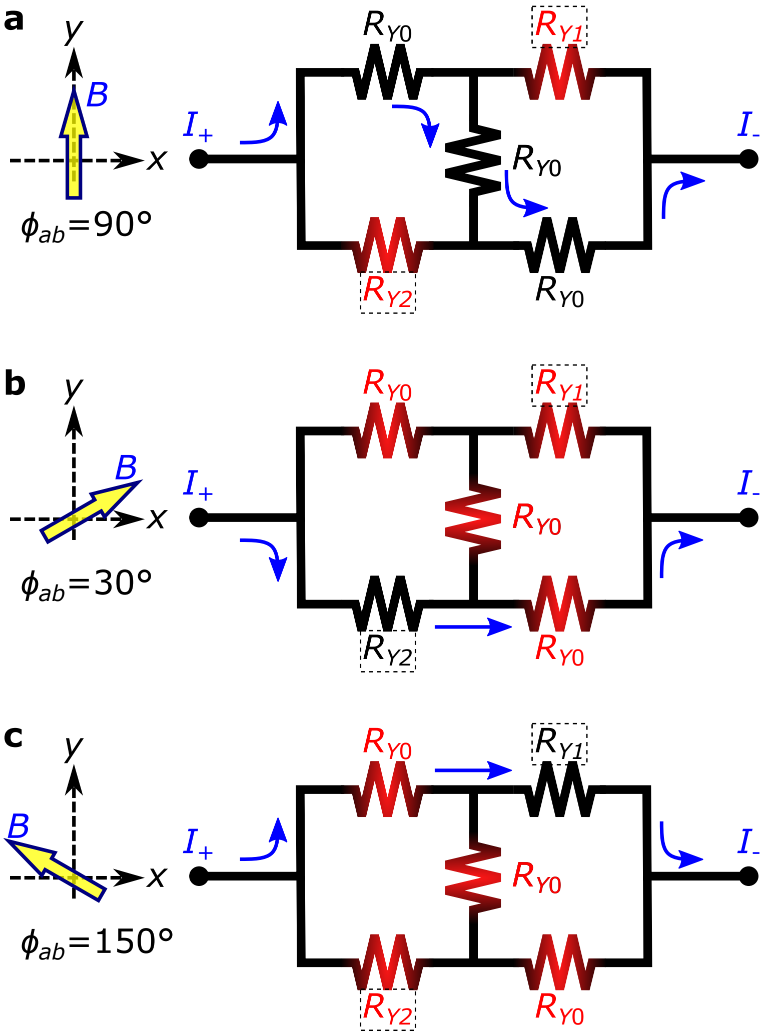

Supplementary Figure 14: Simplified electrical circuit diagrams describing the current path dependence on the direction of the applied field. The black and red color of the resistive element (R) indicate relatively low and high resistance, respectively. The blue arrows indicate the path of the current at each configuration. The yellow arrow indicates the angle (ϕ_{ab}) of the magnetic field (B) relative to the sample's x axis. The three domains are Y_0, Y_1, and Y_2, which are most superconducting at the field angles ±90°, 150°(−30°), and 30°(−150°). **a**, At ϕ_{ab} = 90° R_{Y0} is least resistive and the current passes through the center avoiding R_{Y1} and R_{Y2}. **b**, At ϕ_{ab} = 30° R_{Y2} is least resistive so the current initially avoids R_{Y0} but after the first element the current has to pass through the less resistive path that is R_{Y0} (instead of R_{Y0} and R_{Y1}). **c**, same as (b) except for ϕ_{ab} = 150° and R_{Y1} is initially preferred. The dotted box indicates the minor domain.

Fitting experimental data

We demonstrate validity of the multi-domain circuit model by performing least-squares fitting to the experimental in-plane H_{c2} data defined with the $R/R_n = 95\%$ criterion without and with external strain (blue curves in Supplementary Figures 15 and 16). The fitting was reasonably successful. Note how the multi-domain model accounts for the difference of the peak widths between the domains.

To address another possible interpretation for the change in the H_{c2} anisotropy, namely a strain-induced crossover from a unpinned single nematic domain state to a strongly-pinned single domain state, we discuss the data more thoroughly. We firstly performed a trial fitting with a simple six-fold cosine function $H_{c2}(\phi_{ab}) = H_0 + H_6 \cos(6\phi_{ab})$ that is suggested theoretically for the ideal no-pinning situation[5] (orange curves in the figures). It is clear that, although the six-fold component is most pronounced for H_{c2} using the 95% criteria, the H_{c2} data is impossible to be fitted using the simple six-fold formula. The dominant two-fold behavior is considerably large. Secondly, we find that, with increasing compressive strain, there is a smooth and anisotropic disappearance of the six-fold pattern. In particular, the pair of satellite peaks at $-150°$ & $+30°$ and $-30°$ & $+150°$ have a different strain dependence; as shown in Supplementary Figure 16, the former pair is still evident even under -1.19% strain whereas the latter pair completely disappears. If we were to assume that the six-fold component of H_{c2} is due to a single six-fold domain, then with the application of strain H_{c2} will suddenly exhibit a two-fold behavior and both satellite peaks should be gone simultaneously. However, we do not observe this.

From these considerations as well as from the discussion in the Main Text, we conclude that the multi-domain model provides a more natural interpretation of our experimental data.

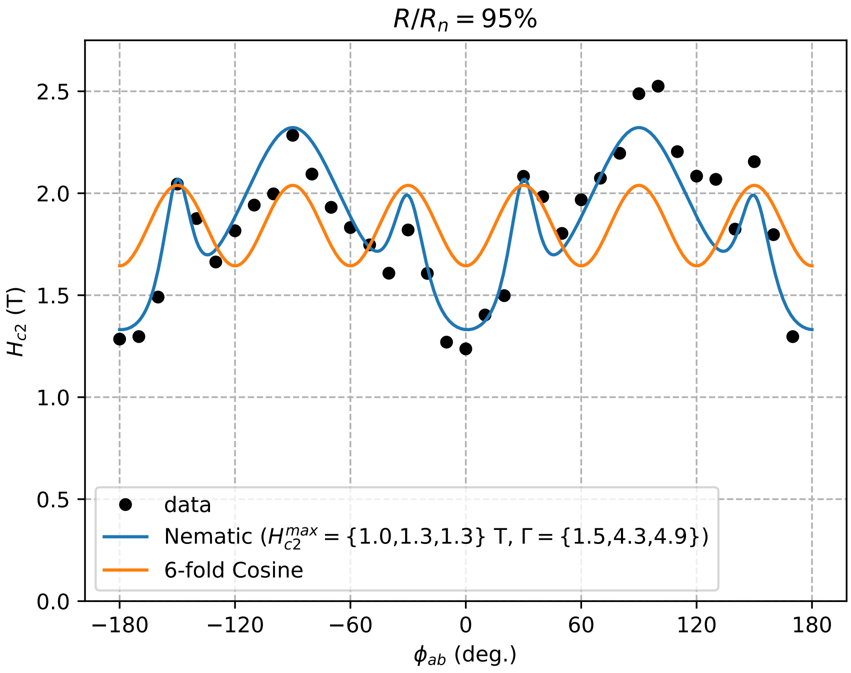

Supplementary Figure 15: In-plane field-angle ϕ_{ab} dependence of H_{c2} defined with the R/R_n = 95% criteria for zero applied strain ($\Delta\varepsilon_{xx}$ = 0). The black points are the measured data and the blue curve is the fitting based on the multi-domain nematic model explained in the text. From the fitting, we obtained fitting parameters H_{c2}^{max} and $\Gamma = H_{c2}^{max}/H_{c2}^{max}$ of each domain as indicated in the figure legend. The orange curve is the result of a trial fitting by using a six-fold sinusoidal function $H_{c2}(\phi_{ab}) = H_0 + H_6 \cos(6\phi_{ab})$ expected for the ideal situation with the absence of pre-existing symmetry breaking field. This result demonstrates that the simple six-fold behavior alone cannot explain the data.

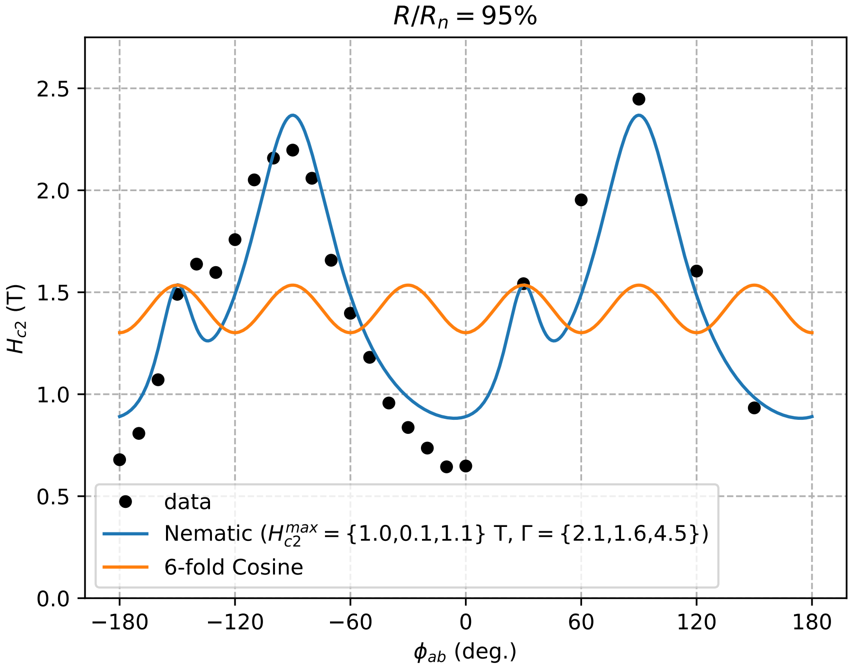

Supplementary Figure 16: Same data as Supplementary Figure 15 but under high compressive strain ($\Delta\varepsilon_{xx}$ = -1.19%). Notice that the shoulder peak at ϕ_{ab}= -30° is completely suppressed, whereas the other shoulder peak at ϕ_{ab}= -150° is still visible even under this high compression. Indeed, the multi-domain model is able to account for the strain induced change.

Dependence on circuit configuration

To check that the simulation results are not specific to our particular choice of circuit configuration, we compare results obtained from various circuit configurations. As shown in Supplementary Figure 17, simulation using very different configurations provides qualitatively similar behavior, as long as the number of the minor domains is smaller than that of the main domain. Note that for the simulation using model 3D (3) at $R/R_n = 5\%$ (Supplementary Figure 17, right-panel, red dotted curve) does not reproduce the two-fold structure for the Y_0 domain. The reason is that it breaks a fundamental assumption in the way the model should be constructed, that is, that the current has to pass through a Y_0 domain. Therefore, as long as this important assumption is maintained we can expect qualitatively good results despite the particularities of the circuit configuration in the model. Thus, our network model indeed provides a simple basis to explain all the observed phenomena including strain dependence.

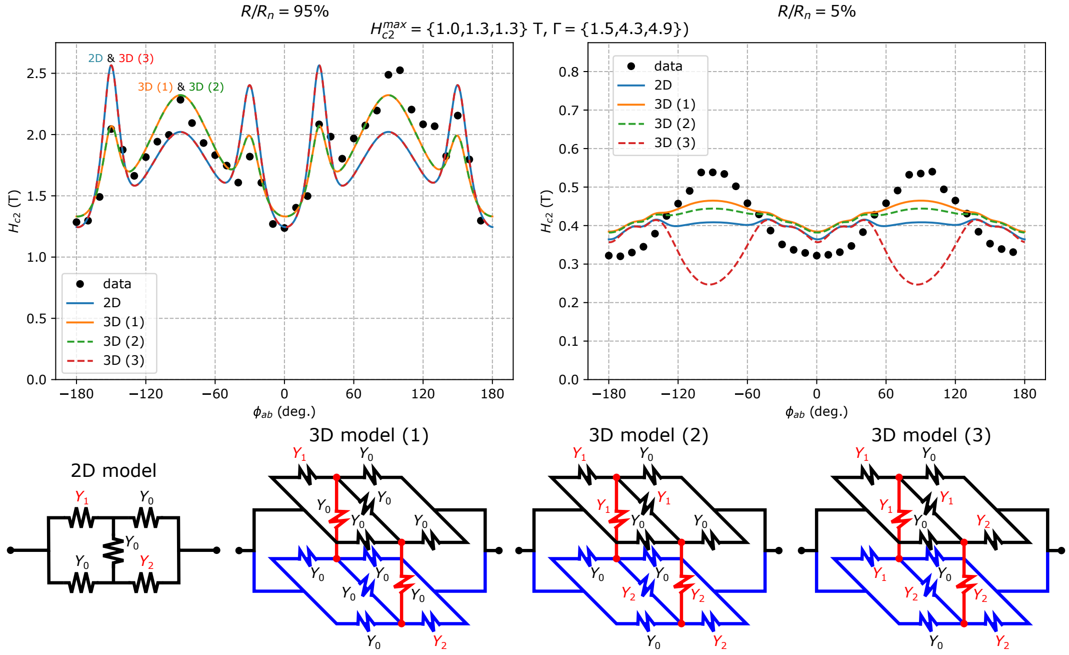

Supplementary Figure 17: Calculated in-plane upper critical field (defined by the 95 and 5% criteria) by the circuit model. Various configurations of nematic domains are illustrated in the bottom of the figure. Experimental data under zero applied strain are also plotted (black circles). For the calculation, we used parameters obtained with the fitting in Supplementary Figure 15 for the standard 3D circuit model (3D model (1); orange curve).

11 *H-T* phase diagrams

To describe the temperature evolution of the strain effect, we show in Supplementary Figure 18 the temperature dependence of H_{c2} determined with various R/R_n criteria along the three principal axes (x, y, z). We also show the in-plane H_{c2} anisotropy ($H_{c2} \parallel y / H_{c2} \parallel x$) under various strain in the bottom panels. With lowering temperature, H_{c2} exhibits linear increase. The in-plane H_{c2} anisotropy ranges 2-3 depending on the H_{c2} criteria. Such a relatively large anisotropy is consistent with previous studies on Sr-doped Bi_2Se_3[4,6]. With an increase of compressive strain, the H_{c2} anisotropy increases for the whole temperature range investigated. Thus, the uniaxial-strain control of nematic superconductivity is achieved irrespective of the temperature range.

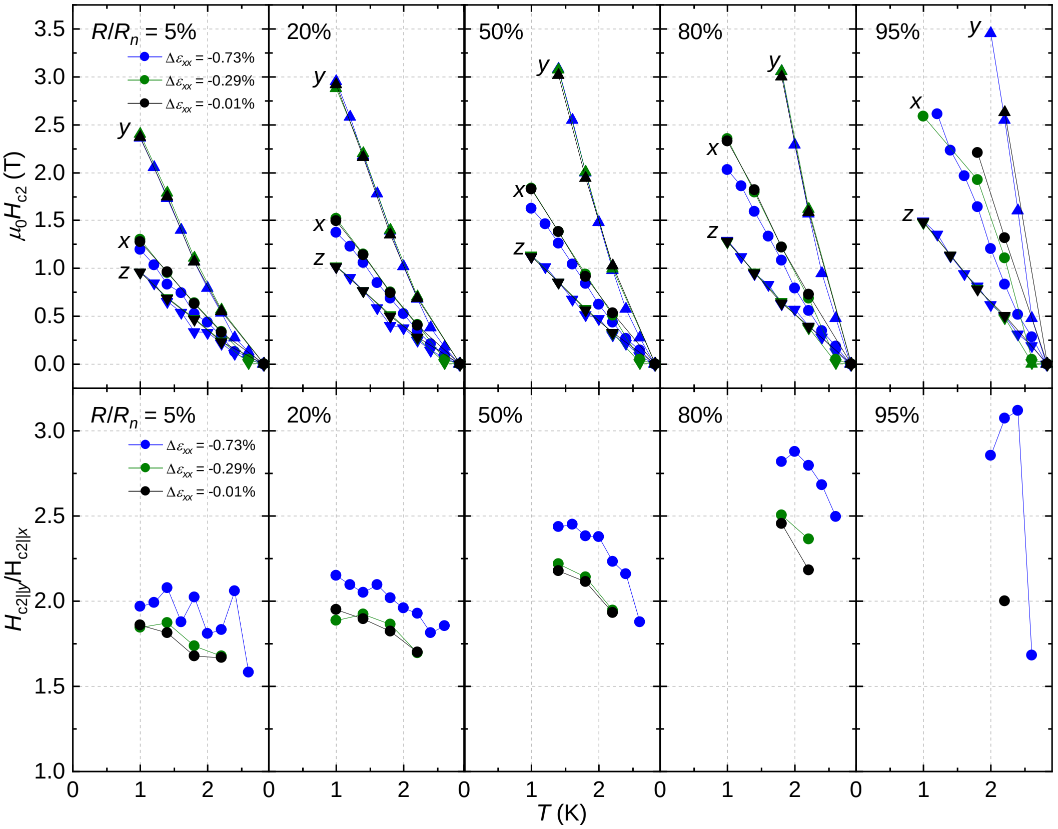

Supplementary Figure 18: Upper critical field H_{c2} (top panels) and in-plane H_{c2} anisotropy (bottom panels) dependence on the temperature for varying applied strain. The colors black, green, and blue correspond to small to larger compressive strains. The numbers in the top corner of each sub-panel indicates the criteria used for determining H_{c2}. Note that at low temperatures there are missing data points due to the magnetoresistance data not having the necessary resistive range for the specified H_{c2} criterion. Source data are provided as a Source Data file.

12 Resistance measured with various current

In order to check that T_c or H_{c2} does not depend on the applied current, we compare resistivity measured with various currents in Supplementary Figure 19. Both in the temperature and field sweeps, the data measured with 100 and 250 μA are almost the same, although there is some suppression of superconductivity with 400 μA. Thus, up to 250 μA, Joule heating or any other current-induced effect should be totally negligible.

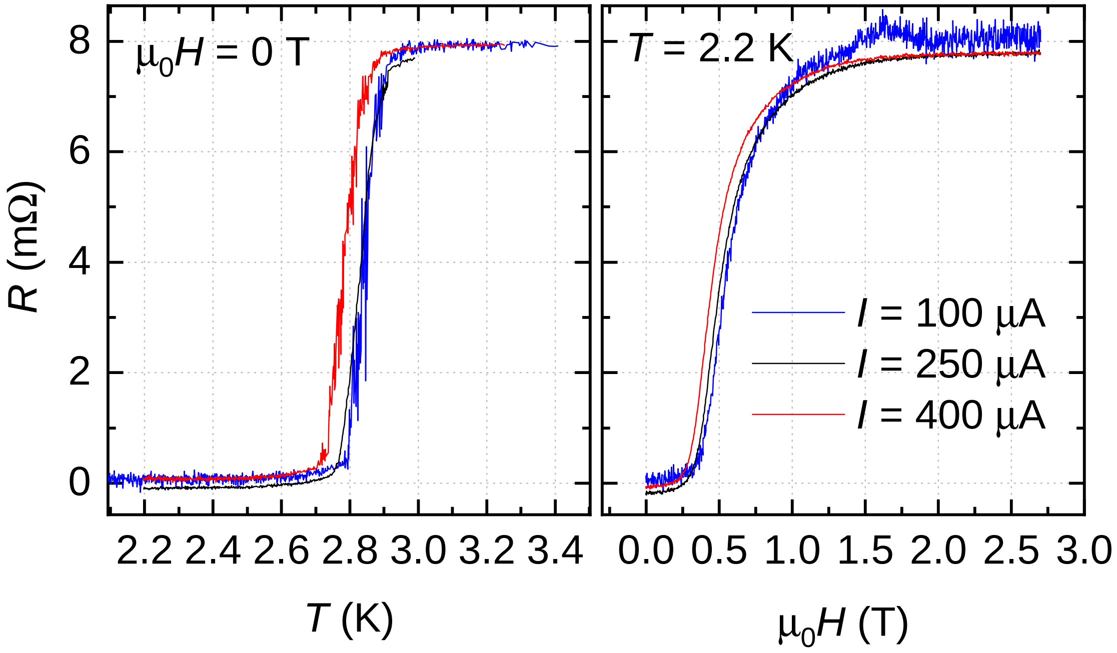

Supplementary Figure 19: Comparison of resistance measured with various currents. The left panel shows the temperature dependence measured at zero field, and the right panel shows the field sweep measured at 2.2 K and for fields nearly parallel to the *x* axis (one of the *a* axes, parallel to the strain direction). For both panels, the data measured with 100 (blue) and 250 μA (black) are almost the same, although there is some suppression of superconductivity under 400 μA. Source data are provided as a Source Data file.

13 Absence of nematicity in the normal-state

In order to check whether nematic behavior exists in the normal-state or not, and to see whether the normal state behavior changes by applied uniaxial strain, we prepared Supplementary Figure 20, comparing field-angle dependence of resistivity at 2.69 T and 2.2 K with and without uniaxial strain. Under this condition, the sample is almost in the normal state for all field directions.

In this figure, we see a weak one-fold (i.e. 360°-periodic) behavior, whose origin is not known. But importantly, the two-fold behavior characterizing normal-state nematicity is absent. Thus, large two-fold behavior in the superconducting state should originate from the emergence of nematic superconductivity. Moreover, it is clear that the uniaxial-strain effects to the field-angle dependence of the normal-state resistivity is practically absent, except for a small constant increase. Thus, the observed strong change of resistivity in the superconducting regime under uniaxial strain should be solely due to the control of nematic superconductivity. Source data are provided as a Source Data file.

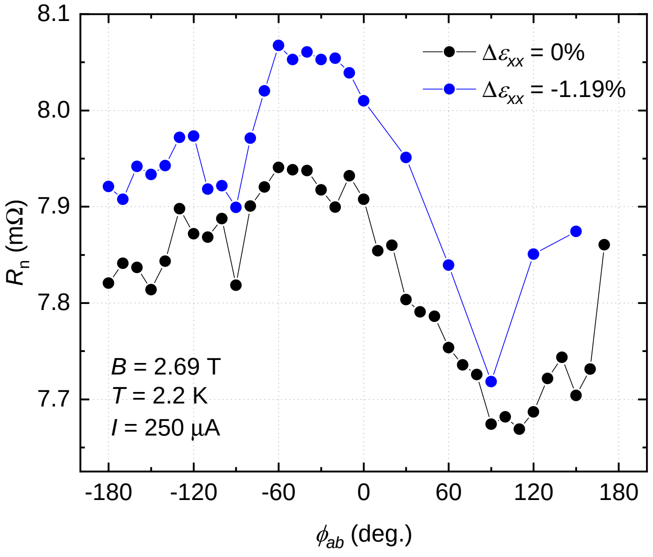

Supplementary Figure 20: Comparison of the angular magnetoresistance in the normal-state measured with and without strain. The data were obtained at 2.2 K and with magnetic field of 2.69 T. Although a weak one-fold change, whose origin is not known, was observed without strain (black data points), two-fold component is almost absent. This fact indicates that the normal-state nematicity is negligibly weak for this sample. Moreover, there is no change in the angular dependence even after application of compressive strain (blue data points).

Supplementary Reference

[1] Nikitin, A. M., Pan, Y., Huang, Y. K., Naka, T. & de Visser, A. High-pressure study of the basal-plane anisotropy of the upper critical field of the topological superconductor $Sr_xBi_2Se_3$. *Phys. Rev. B* **94**, 144516(1–5) (2016).

[2] How, P. T. & Yip, S.-K. Signatures of nematic superconductivity in doped Bi_2Se_3 under applied stress. *Phys. Rev. B* **100**, 134508 (2019).

[3] Deguchi, K., Ishiguro, T. & Maeno, Y. Field-orientation dependent heat capacity measurements at low temperatures with a vector magnet system. *Rev. Sci. Instrum.* **75**, 1188–1193 (2004).

[4] Pan, Y. *et al.* Rotational symmetry breaking in the topological superconductor $Sr_xBi_2Se_3$ probed by upper-critical field experiments. *Sci. Rep.* **6**, 28632(1–7) (2016).

[5] Venderbos, J. W. F., Kozii, V. & Fu, L. Identification of nematic superconductivity from the upper critical field. *Phys. Rev. B* **94**, 094522 (2016).

[6] Du, G. *et al.* Superconductivity with two-fold symmetry in topological superconductor $Sr_xBi_2Se_3$. *Sci. China Phys. Mech. Astron.* **60**, 037411(1–6) (2017).
